# Editorial: Advancements in vibration control for space manipulators: actuators, algorithms, and material innovations

**DOI:** 10.3389/frobt.2025.1681168

**Published:** 2025-09-05

**Authors:** Javad Tayebi, Ti Chen, Xiaofeng Wu, Anand Kumar Mishra

**Affiliations:** 1 State Key Laboratory of Mechanics and Control for Aerospace Structures, Nanjing University of Aeronautics and Astronautics, Nanjing, China; 2 School of Aerospace, Mechanical and Mechatronic Engineering, The University of Sydney, Sydney, NSW, Australia; 3 Department of Mechanical Materials and Aerospace Engineering, West Virginia University, Morgantown, WV, United States

**Keywords:** space manipulators, vibration control, soft robotics, artificial intelligent, on-orbit servicing, smart material, soft griper

Space manipulators play a pivotal role in modern space missions by enabling satellite servicing, debris removal, and planetary exploration. However, their lightweight, long-reach designs and dynamic operational environments introduce significant vibration challenges that can compromise mission success. Addressing these challenges requires a multidisciplinary approach that integrates advancements in actuators, control algorithms, and material science. While conventional actuators (Liu et al., 2021; Tayebi et al., 2022; Mishra et al., 2018) and attitude control strategies (Chen and Shan, 2019; Tayebi et al., 2025; Xie et al., 2025) remain prevalent in spacecraft design, emerging solutions that leverage soft materials and AI-driven control architectures represent a rapidly evolving frontier in vibration mitigation research.

This Research Topic presents the most recent developments in vibration mitigation techniques for space manipulators and includes four important studies resulting from the call. These studies address several aspects of vibration control for space manipulators. Key areas of focus include flexible dynamics estimation via quasi-static approximation, AI-enhanced guidance and control systems, bio-inspired soft actuation, and hybrid soft-rigid grasping architectures.

Accurate measurement of elastic coordinates via sensors has historically posed significant challenges in controlling flexible space manipulators. Patel and Damaren addressed this issue by developing a model-based estimation framework that eliminates the need for direct vibration sensing. They proposed a quasi-static estimator that approximates elastic coordinates with joint torque data, enabling precise end-effector trajectory tracking. Their simulations on single- and two-link manipulators demonstrate robust performance, even with large payloads and model uncertainties.

**FIGURE 1:**
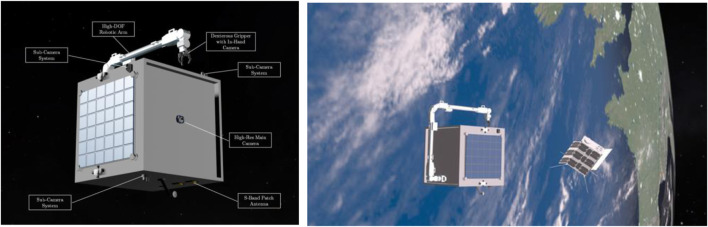
Left: Design of the intelligent orbital service spacecraft. Right: The conceptual mission framework (Hao et al.).

Conventional Guidance, Navigation, and Control (GNC) systems for spacecraft, which are designed for ground-commanded operations with limited autonomy, face significant challenges when adapting to the dynamic demands of on-orbit servicing missions. To address this issue, Hao et al. proposed an AI-enhanced visual GNC system as an intermediate solution between conventional architectures and fully autonomous systems of the future. Their approach combined a deep learning-based algorithm that estimates target pose from 2D images without requiring prior knowledge of the target’s dynamics, and a learning-based motion planner that generates manipulator trajectories while minimizing spacecraft attitude disturbances. The visual GNC system was exemplified through the simulation of a conceptual mission, involving a microsatellite tasked with the on-orbit manipulation of a non-cooperative CubeSat. Figure 1 illustrates the design of the intelligent orbital service spacecraft and outlines the conceptual mission framework for capturing and servicing a non-cooperative target. Their work demonstrates the potential of AI to enhance autonomy in space robotics, particularly for non-cooperative target capture.

**FIGURE 2:**
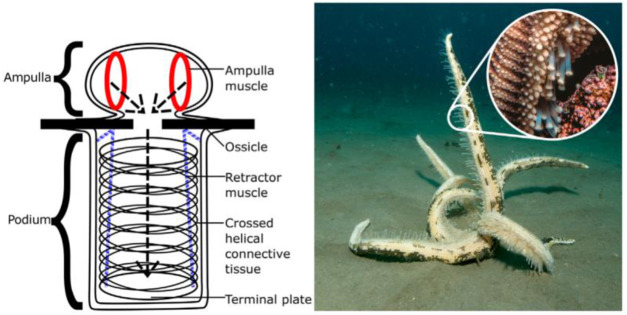
Left: Basic structure of a typical starfish’s podia (tube foot). Right: Close-up of a starfish on the sea floor, with podia in active motion (Ashby et al.).

Actuators play an important role in controlling space manipulators. Ashby et al. introduced bio-inspired soft actuators that use dielectric elastomer transducers (DETs) as lightweight artificial muscles for space applications. Inspired by the starfish podia shown in Figure 2, their inflatable DET-based actuator combines deployable structures with proprioceptive capabilities, enabling compact stowage during launch and adaptive operation in zero-gravity environments. The study highlights the advantages of soft robotics in space, where mass and volume constraints are critical. These actuators show particular promise for applications requiring adaptable, mass-efficient systems in an unstructured orbital environment.

Dontu et al. discussed the development of a hybrid soft gripper designed for delicate object manipulation and validated through real-world robotics competitions. Their vacuum-actuated design integrates soft fingers with rigid components and task-specific modules to balance compliance and precision. By refining the gripper through successive iterations, the work demonstrates the importance of adaptable, hybrid designs for handling diverse objects in unstructured environments.

These studies collectively illustrate three essential developments in space manipulator design through model-based estimation to overcome sensing limitations in orbit, AI-driven autonomy to enable real-time adaptation, and innovations in materials and actuation. Looking ahead, the field must address key challenges in technology readiness levels and orbital validation, particularly regarding soft robotic components in space environments.
